# Entrapment of a metal foreign body in the heart during surgical procedure: A case report and literature review

**DOI:** 10.3389/fsurg.2022.963021

**Published:** 2022-09-20

**Authors:** Fei Wang, Ting Li, Xinwei Yuan, Jiang Hu

**Affiliations:** ^1^ The Orthopaedic Surgery Department of Sichuan Academy of Medical Sciences / Sichuan Provincial People’s Hospital, The Affiliated Hospital of University of Electronic Science and Technology of China, Chengdu, China; ^2^ Department of Postgraduate, Chengdu Medical College, Chengdu, China

**Keywords:** foreign body migration, vascular complication, orthopedic surgery, complication, multidisciplinary collaboration

## Abstract

A rongeur had been used to remove thin bones in both orthopedic surgery and neurosurgery, featured with a tip holding and cutting bone effectively while protecting the underlying instruments. The authors describe a case of a 40-year-old man who proceeded with the second lumbar vertebrae osteotomy and presented to be ankylosing spondylitis with kyphosis and limited mobility for 10 years. During the surgery, we found that the rongeur tip was missing. C-arm fluoroscopy showed the high-density body just in front of the vertebral body intraoperatively. However, the CT scan showed the foreign body migrated to the right auricle of the heart postoperatively. This case is unique in that there was no exact vessel injury detected intraoperatively. There were few reports about the surgical instrument migrating to the heart. Our case showed the rare experience of the function of multidisciplinary collaboration in the migration of foreign bodies in the cervical spinal canal.

## Introduction

A rongeur had been used to remove thin bones in both orthopedic surgery and neurosurgery, featured with a tip holding and cutting bone effectively while protecting the underlying instruments. Our team first reported a rare case where the tip was missing during the surgical procedure and migrated to the heart.

In this case, our team used posterior approach to correct the spine kyphosis deformity. When the instrument is broken and entrapped in the spinal canal during the surgical procedure, immediate removal is not possible. The tip finally migrated to the heart. It is lucky to remove the foreign body successfully without severe outcomes by a 6-month follow-up.

We report this case to warn that retaining foreign objects in the spinal canal is very dangerous. Checking and replacing orthopedic surgery instruments frequently is necessary. In addition, the design for the new rongeur should also consider durability.

## Case report

### Patient presentation

**Figure 1:**
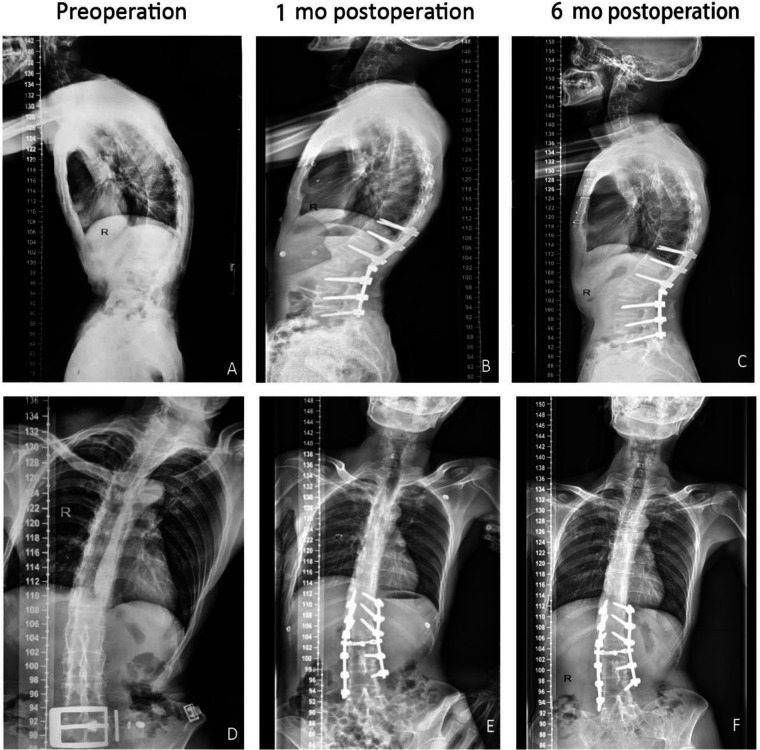
Male, aged 40, ankylosing spondylitis with kyphosis, preoperative whole spine lateral radiographs showed thoracolumbar kyphosis, spine sagittal imbalance (**A**), cobb angle 40° (**D**). The spine sagittal rebalanced (**B,C**) and the Cobb angle corrected to 20° (**E,F**), tested 1 month and 6 months after operation, respectively.

A male patient, aged 40 years, presented with intractable back pain for 10 years. He was not able to lie on the floor for 3 years. As such, he was referred to us for surgery. Whole spine lateral radiographs revealed spine sagittal imbalance (Figures 1A,D), preoperative computed tomography (CT) scan revealed the disappearance of bilateral sacroiliac joint space and the calcification of the vertebral ligaments, while the BLA-27 test was positive. The patient was diagnosed as having ankylosing spondylitis (AS) with kyphosis.

### Clinical procedure

**Figure 2:**
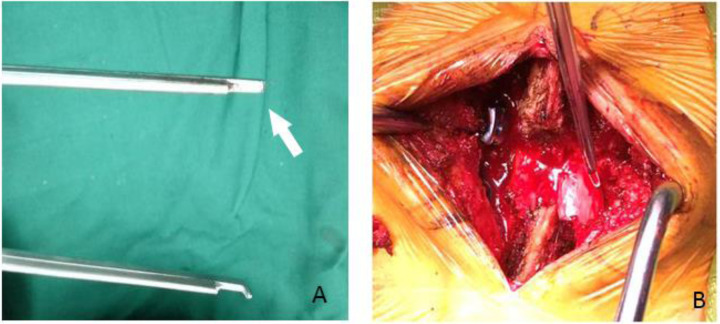
The broken rongeur (arrow) was found in the operation (**A**). The tip was not invisible in the incision (**B**).

**Figure 3:**
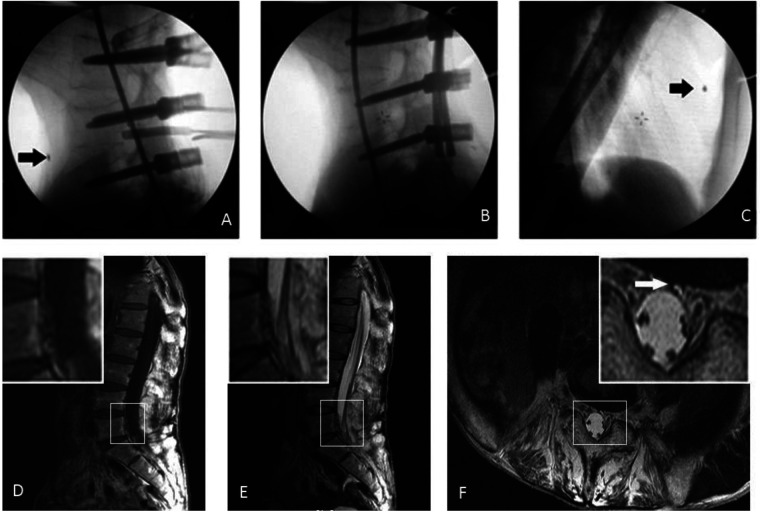
Intraoperative x-ray revealed that the broken metal tip (arrow) was located in the right anterior between the fourth and fifth lumbar vertebrae (**A**). After L2 vertebral body osteotomy, the metal fragment was found to migrate to the heart (**B,C**). MR imaging showed abnormal internal vertebral venous plexus in the dorsal part of the 5th lumbar vertebral body, on T1W (**D**), T2W (**E**), and axial (**F**) images. MR, magnetic resonance.

The patient underwent second lumbar vertebrae osteotomy, decompression, and stabilization in a prone position. During the decompression, the surgeon noted that the Kerrison rongeur was withdrawn without cutting edge and the tip was not visible within the incision (Figure 2). Meanwhile, little blood was encountered and the patient didn't experience hemodynamic compromise. Intraoperative immediate C-arm fluoroscopy manifested that there was a high-density shadow just in the right anterior between the fourth and fifth lumbar vertebrae (Figure 3A). By consultation with cardiac surgeons, vascular surgeons, and gastrointestinal surgeons, immediate anterior exploration was not recommended giving the hemodynamic stability. As per their recommendation, the referring surgeon completed the spine surgery without cerebrospinal fluid leakage while there was 1,000 ml bleeding. At the end of the surgery, we tried to confirm the location of the tip by C-arm fluoroscopy. The tip was found in the left thoracic space between the seventh and eighth rib (Figure 3C). We considered that the tip migrated to the heart through the blood circulation system.

**Figure 4:**
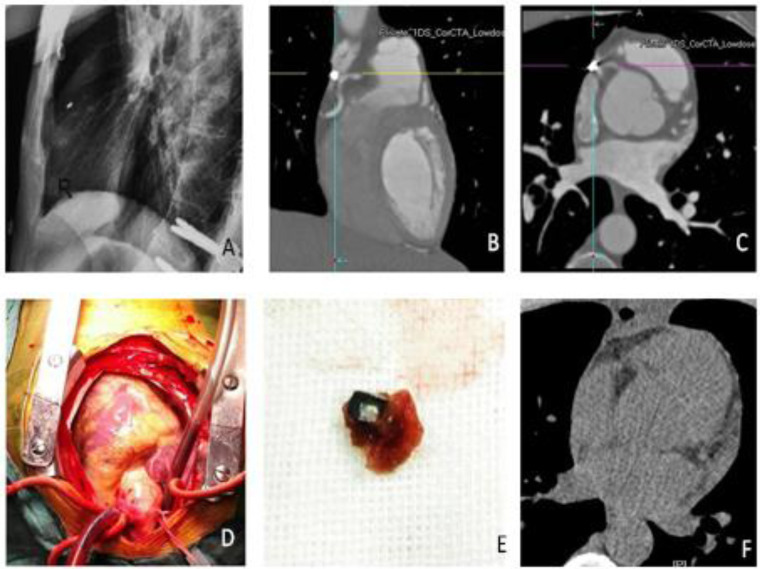
Radiation and dual-source CTA showed that the rongeur tip was in the auricula dextra (**A–C**). The tip was found in the auricula dextra close to the right atrioventricular groove (**D**). Image of the extracted rongeur tip (**E**). CT was used to confirm (**F**).

After surgery, the patient was taken for a dual-source computed tomography angiography (CTA), which revealed that the rongeur tip was located in the auricula dextra (Figure 4A). The patient was transferred to the cardiac surgery department by consulting the cardiologists. Considering the irregular size of the surgical instruments and the difficult technique for retrieval, the cardiac surgery team chose open-heart surgery. The patient underwent open-heart surgery to remove the foreign body (Figure 4). The rongeur tip, about 1 cm in length (Figure 4), was found in the auricula dextra, close to the right atrioventricular groove and right coronary artery.

The low back pain and kyphosis were relieved immediately without any signal of root damage and the patient could lie flat after surgery. Table 1 shows the sagittal score changes of the spine sagittal compared between the preoperative and postoperative stages according to the radiographs (Figure 1). The patient did not show dyspnea, stethalgia, or edema, and Table 2 shows the related data of laboratory inspection after orthopedic surgery and cardiac surgery. The reduction of myoglobin and fibrin and fibrinogen degradation products means that the risk factor is removed successfully, which may induce the thrombus. Echocardiography and coagulation function tests show a good prognosis in the patient's cardiovascular system. The patient returned to normal life 3 months after the surgery. This case was monitored for 6 months. Written informed consent was obtained from the patient for publication of this case report and accompanying images.

**Table 1:** Male, aged 40, ankylosing spondylitis with kyphosis, the spine sagittal score showed the precise value of the spine sagittal according to the radiographs preoperatively, at 1 month, and at 6 months.

	Preoperative	1 month postoperative	6 months postoperative
SVA	11.2 cm	3.2 cm	3.5 cm
PT	33°	12°	14°
PI	53°	53°	52°
LL	35°	62°	60°
PI-LL	18°	−9°	−8°

SVA, sagittal vertical axis; PT, pelvic tilt; PI, pelvic incidence; LL, lumbar lordosis.

**Table 2:** The index of the blood coagulation and cardiomyocyte injury markers were shown preoperatively, at 1 month, and at 3 months after open-heart surgery to guide the treatment and the prognosis.

	Post orthopedic surgery	1 month post cardiac surgery	3 months post cardiac surgery	Standard section
D2	9.05	3.62	1.24	0–0.55
P-FDP	25.2	9.4	7.2	0–5
TPI	0.004	2.237	1.479	0–0.3
CMB	7.3	22.1	12.8	0–6.6
MB	1577.9	428	300.9	0–140.1
BNP	—	11.5	10.2	0–100

The data show the reverse of thrombus and myocardial injury.

D2, D-dimer; P-FDP, fibrin and fibrinogen degradation products; TPI, troponin I; MB, myoglobin; CMB, CK-MB mass; BNP, brain natriuretic peptide.

## Discussion

Physiologists often design instruments with a purpose to make the operation more effective, but the durability of surgical instruments is always neglected. Breakage of instruments during orthopedic procedures reportedly occurs in 0.35% of cases (1). Taking the rongeur as an example, the cutting edge could be designed flatter and smaller in order to make it easy to insert into the layer between the bone and spinal dural. In the operation, the broken tip may be rushed into the spinal canal or even into the abnormal vertebral vessel under pressure. In the spinal canal, the dural sac pulses with the heart beats and the dural pressure change in different postures (2). As such, the spring phenomenon likely pushes the foreign body to migrate or penetrate the intradural space, resulting in encroachment upon the spinal cord or nerve roots (3). Luckily, the patient did not show symptoms of nerve damage after spine surgery. However, while the foreign body is lost in the invisible spine canal and moved in the vertebral canal, it is difficult to withdraw the instrument in the limited cut. Lv et al. had reported the case that keeps the foreign body in the canal without neurological compression (4). However, in our case, the tip is big as 1 cm and retained in auricula dextra, making the situation conditionally to remove it from the body. In spite of the related operative complication, the patient reduced the risk of durative myocardial injury and safety to undertake the MRI test to reduce the radiation exposure caused by the CT scan. Inflammation of tendon and ligament attachment point and ectopic new bone formation were two characteristic pathological changes of AS. On the one hand, by the development of trabecular and cortical osteoporosis and, on the other hand, by the overproduction of cortical bone and entheseal ossification. Therefore, AS patients had aberrant calcification of entheses, which might augment the resistance of bone (5). Because using rongeur orthopedic instruments for a long time will cause certain damage, it is noticed that checking and replacing the orthopedic surgery instruments frequently is necessary, especially for patients with osteosclerosis. In addition, the design for a new rongeur or other orthopedic instruments should also consider durability (6).

Vascular injury is one of the complications, although rare, and the rate ranges from 0.04% to 1.11% in the literature (7, 8). In many studies, the posterior approach was reported safer in reducing the vascular injury to proceed with the interbody fusions. Uribe and Deukmedjian (9) reported 0.1% vascular injury following over 13,000 lateral interbody fusions, while in the paper by Liu (10), it was 0.29% for post-exposure in retrospectively reviewed lumbar spine surgery 1,159 patients. He demonstrated that the incidence was higher in anterior exposure (9.1%) than in posterior exposure. Zahradnik et al. (11) showed a vascular injury in 37 cases of 260 patients during anterior exposure of the thoracolumbar spine (13.8%). Klezl et al. (12) indicated that injury to a major vessel was encountered in 14 (1.11%) cases of 1,262 patients, of which 9 involved an injury to the common iliac vein. As shown in Figure 3, we found that there is a signal of abnormal internal vertebral venous plexus in the dorsal part of the fifth lumbar vertebral body. It explained how the foreign body migrated to the heart while the patient did not experience hemodynamic compromise and no damaged port of the inferior vena cava was found. During the surgery, the blood loss was 1000 ml and 900 ml post operation, which we considered as blood from vertebral bone. “Batson's plexus” was also known as the intraspinal venous plexus. In the spinal canal, the dural pressure changed with heartbeats and respiratory movements, which might allow the tip to enter the “Batson's plexus” with the changes in dural sac pressure. Moreover, we found that there is a signal of abnormal internal vertebral venous plexus in the dorsal part of the fifth lumbar vertebral body, which also might be a significant reason to allow the tip to enter “Batson's plexus.” The tip passes through the Batson plexus to the inferior vena cava and finally to the right atrium. Therefore, we consider that the abnormal internal vertebral venous plexus is responsible for the rare case in the patient with both hemorrhage and foreign body movement.

The guideline of treatment for foreign body migration to the heart was not established due to the complicated situation in these cases. In our case, we found that a foreign body entered the cardiovascular system and reached the right atrium. This is unique. A review of the literature reveals that most of the foreign bodies that migrate to the heart are endovascular devices, such as catheters, guidewires, or pacemakers (13, 14). Naito et al. reported a case of a piece of rubber catheter that migrated to the inferior vena cava during surgery (15). Leonardi and Rivera adopted conservative treatment for their broken cerclage wire in the left heart case considering the high risk of surgical left ventriculotomy associated with searching for the wire that had migrated into the myocardial wall (13). However, the instrument migrating into the heart during spine surgery was less reported so far, and a less-invasive endovascular retrieval would have been preferred in most cases. Bydon et al. recommend percutaneous endovascular or surgical intervention when the diameter of the foreign object exceeds 5 mm, its shape is irregular, or when the patient exhibits clinical symptoms (16). However, the rongeur tip has about 1 cm in length, and its shape is irregular. Minimally invasive surgery might carry greater risks, so we opted for open surgery. The evidence showed that the body migrated to the inferior vena cava from the abnormal internal vertebral venous plexus. In a few reported cases, the retained foreign bodies typically lead to delayed complications within several months or years, including pain, infections, and organ dysfunction. Additionally, migration to the heart of foreign bodies can lead to arrhythmia, perforation, endocarditis, valvular malfunctions, and pericarditis. Some of the complications may happen years later after the initial event (17). On the other hand, some of the complications can be life threatening and be emergency events (18). The foreign body needs to be CT scanned every month while MRI would make the body migrate and cause damage while it migrated into the apparatus; as such, it results in a tumorous tendency. The presentation of foreign bodies varies greatly, and the object that is ingested, the location of the object, whether the event was witnessed, the age of the patient, as well as the timeframe in which ingestion occurred are very important (19). For the same considering factors in our case, the patient was young and in good health, and we have identified what was ingested (a broken rongeur tip) by C-arm. In addition, the location of the object migrated from the Batson's plexus” to the right atrium. Finally, the timeframe in which ingestion occurred is well defined. Therefore, open surgery was chosen as a definitive method for broken rongeur tip retrieval. The patient is lucky to have the foreign body removed without a severe outcome. The metal piece was successfully removed from the auricula dextra by the cardiologist without cardiovascular complications in a 6-month follow-up.

## Conclusion

Broken rongeur tip migration to the heart during spine surgery is a rare complication. Migration can lead to arrhythmia, perforation, endocarditis, valvular malfunctions, and pericarditis. Although the complication is rare, serious consideration should be given to the removal of the broken rongeur tip in the heart space when they are discovered. Our case indicates the necessity of rapid diagnosis and the need for multidisciplinary collaboration and care in the treatment. For young and healthy patients, we suggest surgical intervention to be the treatment.

## Data Availability

The original contributions presented in the study are included in the article/Supplementary Material, further inquiries can be directed to the corresponding author.
